# Decay of infectious adenovirus and coliphages in freshwater habitats is differentially affected by ambient sunlight and the presence of indigenous protozoa communities

**DOI:** 10.1186/s12985-019-1274-x

**Published:** 2020-01-06

**Authors:** Brian R. McMinn, Eric R. Rhodes, Emma M. Huff, Asja Korajkic

**Affiliations:** United States Environmental Protections Agency, Cincinnati, OH 45268 USA

**Keywords:** Coliphage, Adenovirus, Decay, Sunlight, Protozoa

## Abstract

**Background:**

Sanitary quality of recreational waters worldwide is assessed using fecal indicator bacteria (FIB), such as *Escherichia coli* and enterococci. However, fate and transport characteristics of FIB in aquatic habitats can differ from those of viral pathogens which have been identified as main etiologic agents of recreational waterborne illness. Coliphages (bacteriophages infecting *E. coli*) are an attractive alternative to FIB because of their many morphological and structural similarities to viral pathogens.

**Methods:**

In this in situ field study, we used a submersible aquatic mesocosm to compare decay characteristics of somatic and F+ coliphages to those of infectious human adenovirus 2 in a freshwater lake. In addition, we also evaluated the effect of ambient sunlight (and associated UV irradiation) and indigenous protozoan communities on decay of somatic and F+ coliphage, as well as infectious adenovirus.

**Results:**

Our results show that decay of coliphages and adenovirus was similar (*p* = 0.0794), indicating that both of these bacteriophage groups are adequate surrogates for decay of human adenoviruses. Overall, after 8 days the greatest log_10_ reductions were observed when viruses were exposed to a combination of biotic and abiotic factors (2.92 ± 0.39, 4.48 ± 0.38, 3.40 ± 0.19 for somatic coliphages, F+ coliphages and adenovirus, respectively). Both, indigenous protozoa and ambient sunlight, were important contributors to decay of all three viruses, although the magnitude of that effect differed over time and across viral targets.

**Conclusions:**

While all viruses studied decayed significantly faster (*p* < 0.0001) when exposed to ambient sunlight, somatic coliphages were particularly susceptible to sunlight irradiation suggesting a potentially different mechanism of UV damage compared to F+ coliphages and adenoviruses. Presence of indigenous protozoan communities was also a significant contributor (*p* value range: 0.0016 to < 0.0001) to decay of coliphages and adenovirus suggesting that this rarely studied biotic factor is an important driver of viral reductions in freshwater aquatic habitats.

## Introduction

Bacteriophages have a long history of use as model organisms in the realm of molecular biology such as the investigation of the transfer of genes, mechanisms of gene repression and activation, and various gene therapy applications [1]. While bacteriophages have been the subject of research efforts for many years [2, 3], there has been a renewed interest in recent years for practical applications in both public and environmental health arenas. In addition to a growing interest in using bacteriophages as tools to combat antibiotic resistant bacteria [4–7], there has been a recent effort to develop recreational water quality criteria for *Escherichia coli* infecting bacteriophages (i.e. somatic and F+ coliphages) [8].

Coliphages have been used routinely in many monitoring programs (e.g. ground water, aquaculture practices, water reuse, biosolids) [9–11] and rationale for their inclusion in recreational water quality assessment [8, 12] is that their persistence in aquatic habitats can more closely resemble that of viral pathogens because of many morphological and structural similarities [13]. While sanitary quality of recreational waters is routinely assessed through enumeration of fecal indicator bacteria (FIB, such as *E. coli* and enterococci), recent reports identifying viral pathogens as leading causes of recreational waterborne diseases outbreaks [14–17] combined with known differences in fate and transport between FIB and viruses [18–23] highlights the need to evaluate suitability of viral indicators to predict pathogen decay in environmental waters.

Although removal of FIB and viruses through primary and secondary wastewater treatment processes is similar [24–27], viruses are reported to display a greater resilience to wastewater disinfection practices compared to FIB [28–31], allowing them to enter recreational waters through treated wastewater discharge. In contrast, others have shown that reduction of coliphages and viral pathogens through wastewater treatment processes is comparable [18, 32] suggesting that they are similarly affected by exposure to different physical and chemical stressors (e.g. chlorination, UV, peracetic acid, etc). While frequent co-occurrence of coliphages and viral pathogens in environmental waters [33–39], often in the absence of FIB, implies a similar response to various biotic and abiotic environmental stressors, field studies examining this are rare.

Some studies investigating drivers of decay for both coliphage and viral pathogens have suggested that their response to certain environmental stressors is similar. For example, both groups tend to persist longer at lower temperatures [40–43] and in freshwater as compared to marine waters [44–46]. On the other hand, while decay of infectious coliphages is accelerated when exposed to ambient and simulated sunlight [44, 47–51], the response of pathogenic viruses is more ambiguous [50–52] and possibly influenced by laboratory measurement strategies (infectious viruses enumerated on mammalian cell cultures versus molecular approaches such as qPCR enumerating viral nucleic acids) [52, 53]. Even less is known about the potential effect of biotic stressors, such as protozoan predation, on decay of both coliphages and viral pathogens. Greater decay in the presence of indigenous microbiota has been demonstrated for FIB and some bacterial pathogens [54–57], but analogous information is needed for viruses.

Factors impacting viral persistence in natural systems are difficult to simulate, necessitating an experimental design that closely mimics natural conditions. To address these research gaps, we employed a submersible aquatic mesocosm (SAM) to study decay of coliphages (somatic and F+) and infectious adenoviruses in a freshwater lake under in situ conditions. We also investigated the effect of indigenous protozoan communities and ambient sunlight to better understand the biotic and abiotic factors impacting the decay of viruses in natural aquatic environment.

## Materials and methods

### Experimental design

Ambient water (~ 15 L) was collected from William H. Harsha Lake (Batavia, OH: 39.0252°N, − 84.1303° W). Immediately after collection, 50% of the sample was passed through a 0.80 μm filter to remove indigenous protozoa. Filtration of water to remove protozoa is a common method and more effective than other techniques such as chemical treatments [58–62]. To minimize any changes in microbial populations, filtered and unfiltered water was stored in dark at 4 °C until the beginning of the experiment (< 48 h). In order to closely mimic ambient conditions by in situ incubation (at William H. Harsha Lake), a SAM was used to conduct the study. The SAM was constructed as previously described [54, 63–66] and samples were contained using regenerated cellulose dialysis bags (75 mm flat width, 13–14 kD pore size molecular weight cut-off, Spectrum Labs, Rancho Dominguez, CA). The first day of the experiment, both filtered and unfiltered ambient water was spiked with somatic and F+ coliphages and adenovirus and stirred for 15 min to ensure proper distribution of the spikes within the sample. Measured portions of either spiked filtered or spiked unfiltered ambient water (200 mL) were used to fill each dialysis bag. One half of the dialysis bags containing each water type was attached at the top portion (approximately 2–5 cm below the water surface for the light exposure treatment), while the other half was placed at the bottom portion (approximately 25–30 cm below the water surface underneath the heavy-duty black plastic tarp for shaded treatment). For the study, four different treatments were as follows: A: exposure to ambient sunlight and indigenous microbiota including protozoa (top level, unfiltered water), B: exposure to indigenous microbiota including protozoa, (bottom level, unfiltered water), C: exposure to ambient sunlight only (top level, filtered water) and D: exposure to neither variable (bottom level, filtered water). During each sampling event, triplicate dialysis bags for each treatment were processed for the enumeration of somatic and F+ coliphages, as well as infectious adenovirus (as described below). Concentrations of all viruses were obtained immediately after the inoculum preparation (day 0) and after one and eight days of exposure. Two additional time points (days 3 and 5) were processed for both coliphage types.

### Bacteriophage enumeration

Somatic and F+ coliphage were enumerated using double agar layer (DAL) procedure, as previously described [67]. If necessary, decimal dilution series were prepared using 1X phosphate buffered saline solution (PBS: 0.0425 g/L KH_2_PO_4_ and 0.4055 g/L of MgCl_2_; pH 7.2 Sigma Aldrich, St. Louis, MO). Briefly, 1 mL of sample was added to 5 mL of “molten” top tryptic soy agar (TSA) layer (0.7% agar) containing 0.1% of appropriate antibiotic stock solution (100 μg/ mL nalidixic acid for somatic and 15 μg/ mL streptomycin/ampicillin for F+ coliphage) (Fisher Scientific, Waltham, MA), followed by addition of 200 μl of appropriate *E. coli* host (CN-13 ATCC#700609 [somatic] of F_amp_ ATCC#700891 [F+], American Type Culture Collection, Manassas, VA) in mid-log growth phase. The soft agar overlay mixture was mixed and poured on bottom agar TSA plates (1.5% agar and containing 0.1% of appropriate antibiotic stock solution). Plates were incubated at 37 °C for 16–18 h. The following day characteristic plaque forming units (PFU) for each coliphage type were enumerated and data were expressed as PFU per 1 ml. Method blank (sample substituted with 1X PBS) and media sterility negative controls were performed on each day of the experiment. For the duration of the study, no plaques were observed on any of the negative controls indicating absence of contamination.

### Adenovirus enumeration

Human lung cells (A549, ATCC® CCL-185) were propagated in Dulbecco’s Minimum Essential Medium (DMEM high glucose with HEPES, Greiner, Monroe, NC) supplemented with 10% fetal calf serum (Fisher Scientific) and 1% sodium pyruvate (Fisher Scientific) under 5% CO_2_ atmosphere and at 37 °C. Test cultures of A549 cells were planted and grown to 90% confluency for 4 days in 25 cm^2^ filter capped flasks at 37 °C using a maintenance medium (as described above) except: 1) the addition of antibiotic-antimycotic solution (1% v/v, Fisher Scientific) and 2) reduced fetal calf serum amount of 2% v/v. Prior to inoculation with samples, test cultures were washed with 10 mL of Earle’s Balanced Salt Solution per flask (EBSS, Fisher Scientific) supplemented with 1% antibiotic-antimycotic solution. Decimal dilution series of samples were created using 1X PBS and five replicate flasks per dilution were utilized. In addition, ten negative control flasks (containing 10 mL of 1X PBS instead of the sample) were ran with each sample batch. Following inoculation, flasks were placed on a rocker for 90 min to allow for viral attachment/infection to occur. Flasks were then supplemented with 10 mL of maintenance medium and incubated at 37 °C for 3 weeks [68]. During the incubation time, flasks were examined weekly for the formation of cytopathic effects (CPE). Concentrations of adenovirus were estimated using EPA’s Most Probable Number (MPN) calculator Version 2.0 (https://cfpub.epa.gov/si/si_public_record_report.cfm?Lab=NERL&dirEntryId=309398). Resulted are reported as MPN per 1 mL.

### Virus spike preparation

Primary treated wastewater was collected from a local wastewater treatment plant and used as a source of somatic and F+ coliphages. Briefly, 10 mL of wastewater was syringe filtered (0.22 μm pore size) and added to 100 mL of mid-log culture of appropriate *E. coli* host. The inoculated host cultures were incubated at 37 °C for 16-18 h, followed by centrifugation (3800 x g, 15 min) and syringe filtration (0.22 μm pore size). The resulting coliphage stocks were titered using DAL as described above and stored in dark at 4 °C until the beginning of the experiment (~ 24 h).

Human adenovirus 2 (ATCC® VR-846) was obtained from ATCC and propagated in A549 cells to generate higher titers. Briefly, A549 cells were infected with adenovirus as described above for the samples. Following the development of CPE (typically in < a week), cells underwent three freeze-thaw cycles, followed by centrifugation at 2500 x g for 30 min to pellet cellular debris. The supernatant was syringe filtered (0.22 μm pore size), titered (as described above for cell culture samples) and stored in dark at 4 °C until the beginning of the experiment.

### Visible light and temperature measurements

For the duration of the study, hourly light intensity (lum/ft^2^) and temperature (°C) measurements were recorded at both upper and lower SAM levels using HOBO® UA 002–08 data loggers (Onset Computer Corporation, Bourne, MA). The temperature at the top level (16.67 ± 1.18 °C) was slightly higher (paired t-test, *p* = 0.0394) compared to the bottom level 16.59 ± 0.88 °C), but the light intensity was considerably greater (paired t-test, *p* < 0.0001) at the top (54.34 ± 146.73 lum/ft^2^) compared to the bottom level (9.47 ± 19.15 lum/ft^2^).

### Data analysis

All concentration data were log_10_ transformed prior to data analyses. Log_10_ reductions were calculated by subtracting concentrations obtained on day “n” (where “n” represents days 1, 3, 5 or 8) from concentration at the beginning of the experiment (day 0). GraphPad Prism version 7.01 (GraphPad Software, La Jolla, CA) was used to conduct a two-way analysis of variance (ANOVA with interactions) with Tukey’s multiple comparison test to evaluate the effects of two factors (indigenous microbiota including protozoa and sunlight) on decay. This software was also used to conduct the paired t-tests, one-way ANOVA and Pearson product momentum correlation to assess significant differences in light temperature measurements, across different virus measurements and to identify potential correlations trends in decay patterns, respectively.

## Results

### Decay characteristics in freshwater environment

**Fig. 1:**
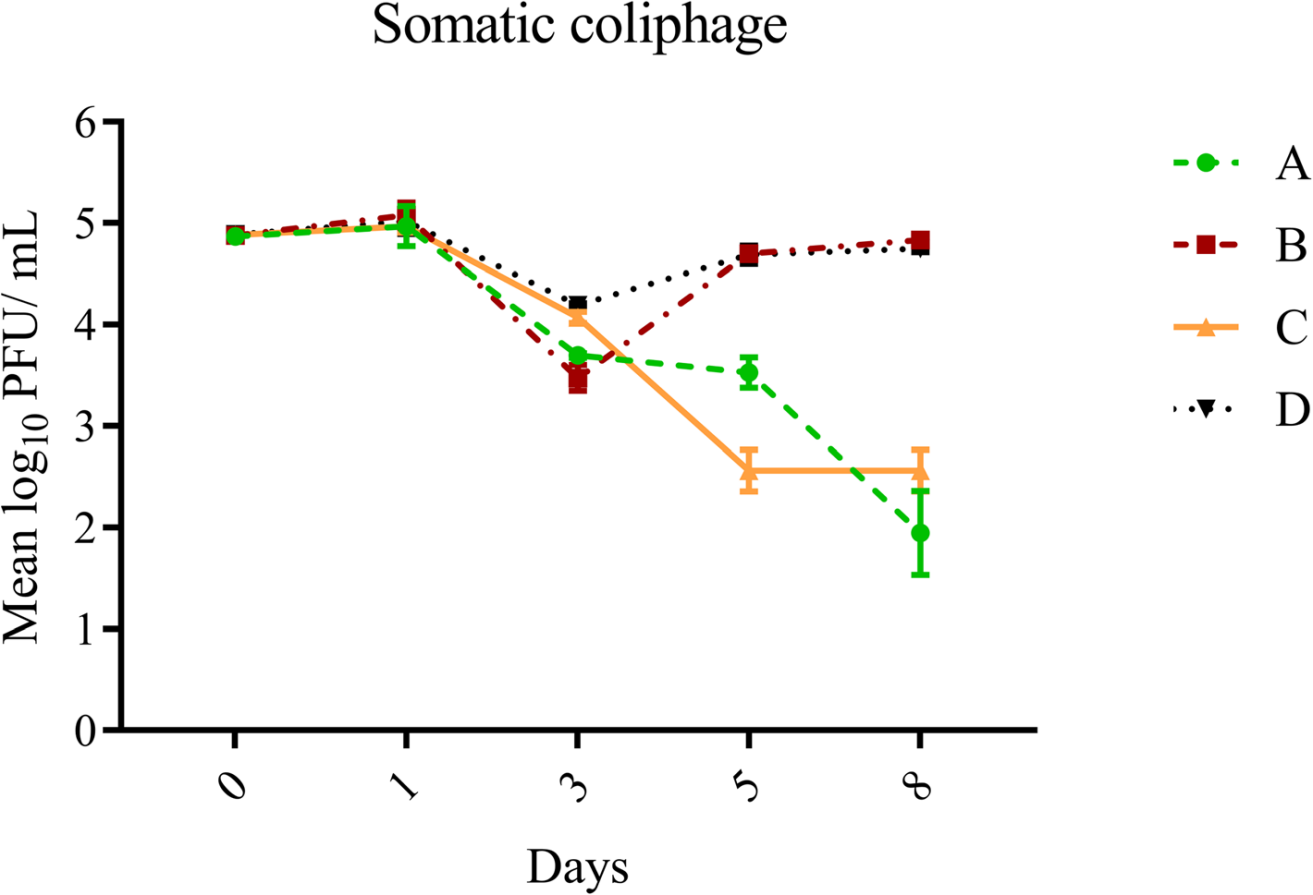
Effect of treatment variables on changes in somatic coliphage concentrations over time. Error bars represent standard deviation. Treatments: A (exposure to sunlight and indigenous microbiota including protozoa), B (exposure to only indigenous microbiota including protozoa), C (exposure to sunlight only), D (exposure to neither)

**Fig. 2:**
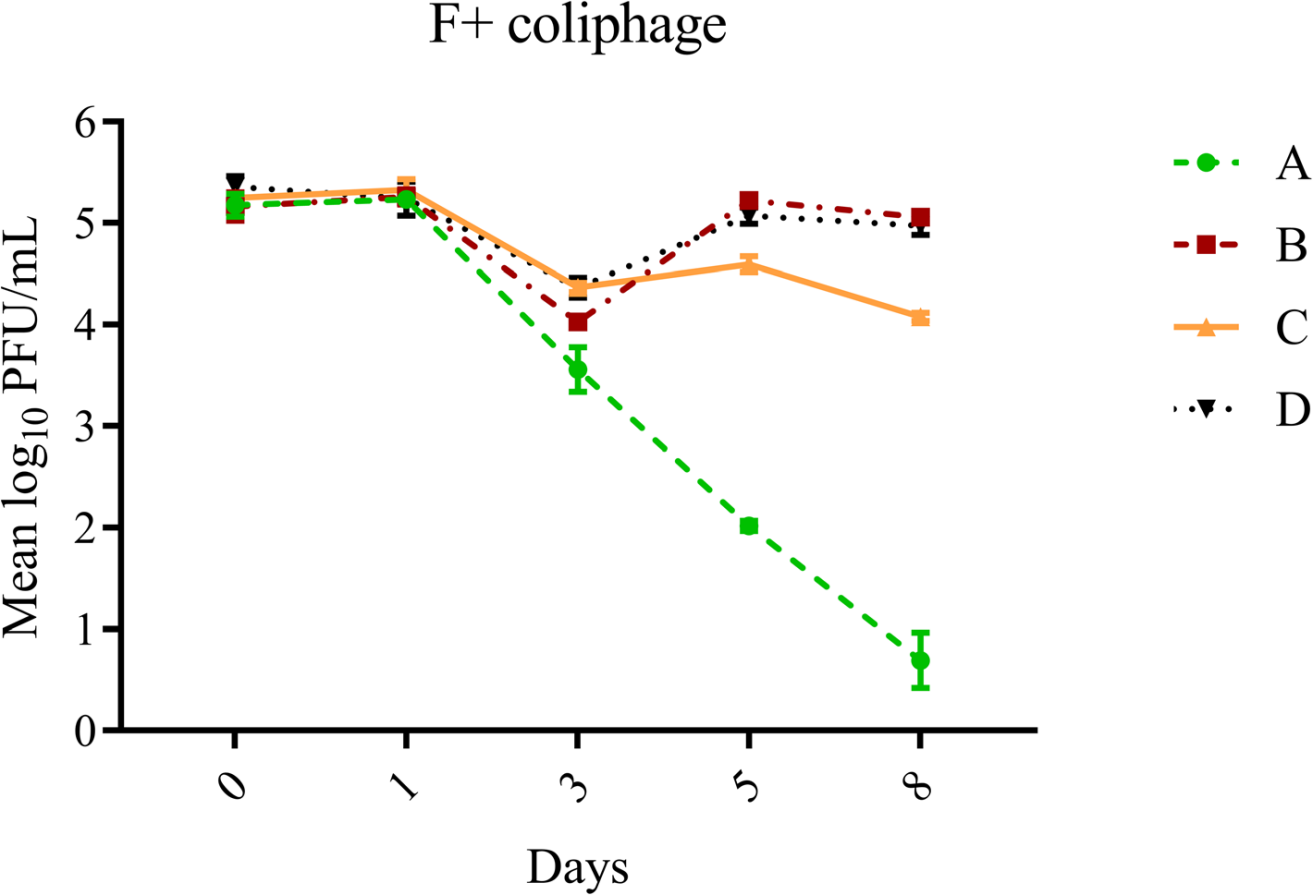
Effect of treatment variables on changes in F+ coliphage concentrations over time. Error bars represent standard deviation. Treatments: A (exposure to sunlight and indigenous microbiota including protozoa), B (exposure to only indigenous microbiota including protozoa), C (exposure to sunlight only), D (exposure to neither)

**Fig. 3:**
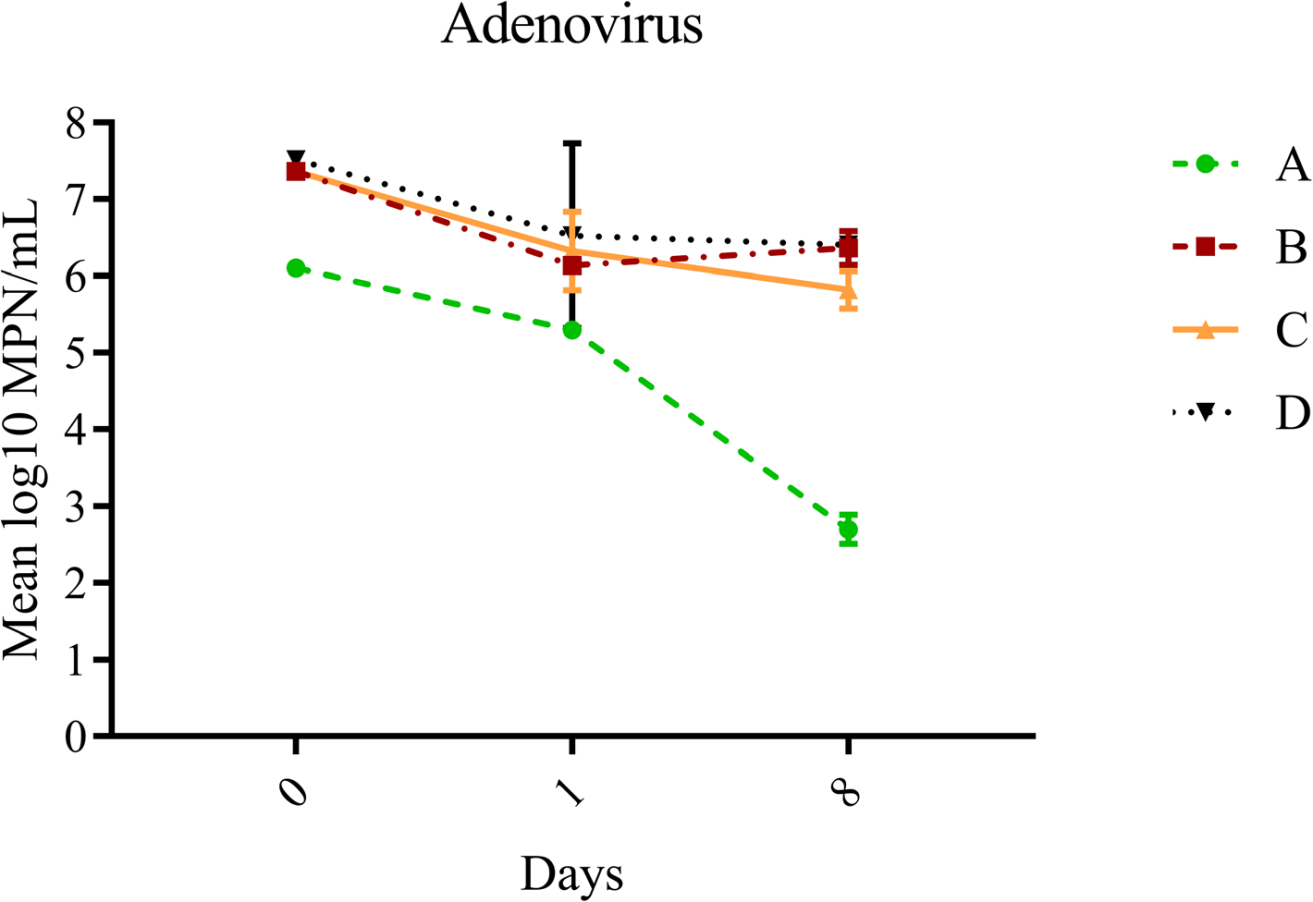
Effect of treatment variables on changes in adenovirus concentrations over time. Error bars represent standard deviation. Treatments: A (exposure to sunlight and indigenous microbiota including protozoa), B (exposure to only indigenous microbiota including protozoa), C (exposure to sunlight only), D (exposure to neither)

Overall, average log_10_ reduction on days one and eight for all treatments was greatest for adenovirus (1.48 ± 0.99), followed by F+ (0.79 ± 1.53) and somatic (0.61 ± 1.21) coliphages, although these differences were not statistically significant (*p* = 0.0794). After 8 days, exposure to sunlight and indigenous microbiota (Treatment A) resulted in the greatest decay for all three organisms (log_10_ reductions of 2.92 ± 0.39, 4.48 ± 0.38, 3.41 ± 0.19 for somatic coliphages, F+ coliphages and adenovirus, respectively) (Table 1, Figs 1, 2 and 3). Exposure to sunlight only (Treatment C) resulted in log_10_ reductions of 2.31 ± 0.20, 1.17 ± 0.01 and 1.54 ± 0.24 for somatic coliphages, F+ coliphages and adenovirus respectively, while shaded treatments (Treatments B and D) typically yielded the least decay (log_10_ range: 0.05–1.11) (Table 1, Figs 1, 2 and 3). Decay pattern of all viruses was strongly correlated (r^2^ range: 0.754–0.881, *p* value range: 0.0002 - < 0.0001), although it was the most noticeable for F+ coliphage and adenoviruses (*r*^2^ = 0.881, *p* < 0.0001).

**Table 1:** Log_10_ reduction values for somatic coliphage, F+ coliphage and adenovirus. Treatments: A (exposure to sunlight and indigenous microbiota including protozoa), B (exposure to only indigenous microbiota including protozoa), C (exposure to sunlight only), D (exposure to neither)

Organism	Time point	Treatment			
		A	B	C	D
Somatic	1	−0.10 ± 0.17	−0.20 ± 0.09	−0.09 ± 0.05	−0.13 ± 0.13
	3	1.17 ± 0.04	1.40 ± 0.12	0.81 ± 0.05	0.70 ± 0.04
	5	1.34 ± 0.17	0.18 ± 0.02	1.66 ± 0.19	0.20 ± 0.13
	8	2.92 ± 0.39	0.05 ± 0.03	2.32 ± 0.20	0.14 ± 0.00
F+	1	−0.06 ± 0.10	−0.10 ± 0.21	−0.08 ± 0.08	0.12 ± 0.18
	3	1.62 ± 0.22	1.14 ± 0.17	0.88 ± 0.09	1.00 ± 0.14
	5	3.16 ± 0.11	−0.06 ± 0.18	0.65 ± 0.11	0.29 ± 0.15
	8	4.48 ± 0.38	0.19 ± 0.05	1.17 ± 0.01	0.39 ± 0.15
Adenovirus	1	0.81 ± 0.00	1.23^a^	1.04 ± 0.51	0.99 ± 1.20
	8	3.41 ± 0.19	1.00 ± 0.22	1.54 ± 0.24	1.11 ± 0.10

^a^Single sample

### Effect of experimental variables on decay of somatic coliphages

After 1 day of exposure, neither variable (ambient sunlight and protozoan grazing) had a significant effect on decay, and log_10_ reductions for all treatments were negligible. Over the course of the next 48 h (day three), unfiltered treatments containing indigenous protozoa exhibited greater decay (log_10_ reduction values: 1.17 ± 0.04 and 1.40 ± 0.12; (Treatments A and B, respectively) compared to filtered treatments (< 1 log_10_ reduction; Treatments C and D) (Table 1, Fig. 2). While presence of protozoa was the only significant variable affecting decay at day three (Table 2) and contributing ~ 86% to variation in the data set, the interaction between variables was also significant (*p* = 0.0026) indicating that the effect of protozoa was dependent on sunlight exposure (Table 2). Forty-eight hours later (day five), effects of protozoa began to diminish (Fig. 1, Table 1), and sunlight irradiation became the dominant factor affecting the decay (log_10_ reduction values: 1.34 ± 0.17 and 1.66 ± 0.19 and for Treatments A and C, respectively) and contributing ~ 94% to the observed variation in decay (Table 2). At day eight, which was the final time point, solar irradiation continued to be the dominant factor (~ 95% contribution to the observed variability in the dataset) and was the only influential variable (Table 2) causing the log_10_ reduction values of 2.92 ± 0.39 and 2.32 ± 0.20 for Treatments A and C, respectively (Table 1, Fig. 1).

**Table 2:** Two-way ANOVA with Tukey’s post-hoc test of treatment effects during each time point. Statistically significant values are bolded

Organism	Time point (days)	Sun		Protozoa		Interaction	
		*p* value	% contribution^a^	*p* value	% contribution^a^	*p* value	% contribution^a^
Somatic	1	0.3330	11.03	0.5509	4.027	0.6883	1.799
	3	0.1793	1.045	**< 0.0001**	**86.18**	**0.0026**	**8.913**
	5	**< 0.0001**	**94.13**	0.0793	1.552	0.1092	1.248
	8	**< 0.0001**	**95.28**	0.0796	0.9771	**0.0259**	**1.805**
F+	1	0.3738	7.364	0.2977	10.3	0.2033	15.93
	3	0.0917	8.448	**0.0016**	**49.88**	**0.0130**	**23.26**
	5	**< 0.0001**	**49.63**	**< 0.0001**	**18.06**	**< 0.0001**	**31.47**
	8	**< 0.0001**	**50.31**	**< 0.0001**	**18.91**	**< 0.0001**	**24.13**
Adenovirus	1	0.7955	1.843	0.9957	0.0008	0.7477	2.853
	8	**< 0.0001**	**49.23**	**0.0001**	**18.86**	**< 0.0001**	**23.89**

^a^Percent contribution to variability in the dataset

All of the boldface entries in Table 2 are significant at an alpha level of 0.05

### Effect of experimental variables on decay of F+ coliphages

Similar to somatic coliphages, decay of F+ coliphages was minimal within the first 24 h of exposure and neither variable had significant effect on decay (Tables 1 and 2, Fig. 2). At day three, 48 h later, exposure to indigenous microbiota had a significant effect on decay contributing ~ 50% to the observed variations in the data set with minimal interactions (Table 2).

The greatest decay occurred in Treatment A (exposure to sunlight and biota; log_10_ 1.62 ± 0.22), followed by Treatment B (exposure to biota only; log_10_ 1.14 ± 0.17) and finally Treatments C and D (exposure to sun only and exposure to neither variable; < 1.00 log_10_ reduction each) (Table 1, Fig. 2). Presence of protozoa continued to significantly affect the decay of F+ coliphages at day five, but its contribution to the variability was less (~ 18%). Exposure to sunlight became a dominant significant variable on day five, contributing nearly 50% to the observed variation in the dataset (Table 2). Overall, the greatest decay occurred for the treatment containing both, indigenous protozoa and sunlight (log_10_ reduction: 3.16 ± 0.11), indicating that the effect of variables was co-dependent (Table 1). During the final time point (day eight), the effect of both variables (as well as their interaction) continued to be statistically significant and their contribution to the decay remained similar to that at day five (*p* = 0.0001; Table 2). Decay continued to be the most pronounced when F+ coliphage were exposed to both variables (log_10_ reduction Treatment A: 4.48 ± 0.38), followed by exposure to sunlight only (log_10_ reduction Treatment C: 1.43 ± 0.10) while the decay in the remaining two treatments was negligible (Table 1, Fig. 2).

### Effect of experimental variables on decay of infectious adenovirus 2

Although decay data for adenovirus is limited, similar to both coliphages, neither variable had a significant effect on decay within the first 24 h of exposure (Table 2, Fig. 3). Over the course of 8 days, both sunlight and indigenous microbiota were significant factors contributing to the decay of adenovirus (Table 2, Fig. 3). Sunlight was more important variable contributing nearly 50% to the observed variations in the data set, followed by interactions between the variables (~ 24%) and indigenous biota (~ 19%) (Table 2). In sunlight treatments, adenovirus reduction in presence of protozoa (Treatment A) was approximately 2 log_10_ greater compared to the reductions in their absence (Treatment C) (3.41 ± 0.19 vs 1.54 ± 0.24) (Table 1, Fig. 3), whereas the reduction in dark treatments was ~ 1 log_10._

## Discussion

Recent reports indicate that the majority of recreational waterborne illnesses are caused by viral pathogens [14–17]. As a result, routine monitoring of recreational waters with FIB may not adequately represent viral pathogen presence due [69], at least in part, to different decay trends between these two groups [18]. Coliphages are an attractive alternative because they have similar morphological characteristics to those of many pathogenic viruses suggesting they can better mimic their survival compared to FIB [70, 71]. Earlier studies reported that somatic and F+ coliphages were adequate surrogates for fate and transport of poliovirus [72] and noroviruses [73], respectively. In this in situ field study we used a SAM to compare the decay characteristics of somatic and F+ coliphages to that of infectious adenovirus and to evaluate the effect of ambient sunlight and indigenous protozoan communities on their decay.

The effect of ambient sunlight (and associated UV-A and UV-B radiation) on decay of various indicators and pathogens is likely one of the most commonly studied abiotic environmental factors [74]. Briefly, the damage caused by ambient sunlight can be classified into two categories, direct and indirect based on the mode of action [75]. Direct damage is caused by UV-B and it results in a formation of pyrimidine dimers, while UV-A causes indirect, photooxidative damage which can be exogenous or endogenous depending on the location of free radicals and reactive oxygen species [75]. Earlier studies noted that the decay of infectious adenoviruses exposed to natural and simulated sunlight [50, 76, 77] was typically greater than their corresponding qPCR signal [52, 53, 78] in both marine and freshwaters. Similar findings were observed for infectious somatic and F+ coliphages [44, 50, 66, 76]. We also noted a strong influence of ambient sunlight on decay of infectious coliphages and adenovirus 2, especially after 5 days of exposure, although it is worth noting that we used a singular, laboratory propagated strain of adenovirus and that indigenous, environmental strains may exhibit greater resilience [79–81]. Furthermore, the effect of ambient sunlight was more pronounced for somatic coliphages, compared to F+ coliphages and adenoviruses. This is consistent with previous studies [50, 66, 76, 82] reporting a greater susceptibility of somatic coliphages to sunlight compared to other viral groups. While exposure to both UV-A and UV-B spectrum is detrimental, earlier studies investigating the mechanism of sunlight action, suggest that indirect, photooxidative damage may be the primary mechanism for adenovirus and F+ coliphages [44, 51, 76], while direct damage caused by UV-B is the dominant mechanism for somatic coliphages [44, 76]. However, additional controlled, laboratory based mechanistic studies are needed to confirm that the greater susceptibility of somatic coliphages, as compared to F+ coliphages and adenoviruses, to sunlight is due to differential decay modes of action.

Ciliates and heterotrophic nanoflagellates are effective grazers in the water column [83] and an important part of microbial food webs in many different aquatic habitats [84]. The abundance of these two groups in oligo-mesotrophic waters, such as William H. Harsha Lake is typically estimated to be between 10^2^ and 10^4^ cells per mL [85, 86]. While the effects of protozoan predation have been demonstrated for FIB and other bacteria in field studies [54–56, 66], the role biotic interactions play in decay of viruses is rarely explored. Laboratory feeding experiments demonstrated uptake of various adenoviruses (serotypes 2, 11 and 41) by ciliate *Euplotes octocarinatus* [87] and a free-living amoeba, *Acanthamoeba castellanii* [88], as well as adsorption of adenovirus 2 on the surface of wild ciliates isolated from active sludges of a wastewater treatment plant [87]. However, direct immunofluorescent antibody techniques were used to detect adenoviruses inside and on the surface of the protozoan cells [87, 88] and it is unclear whether the viruses were infectious. Laboratory decay studies conducted in the dark and in the absence of indigenous microbiota (autoclaved ground and river water) noted extended persistence of infectious adenovirus 2 and 41 [89, 90], but the faster decay of infectious poliovirus type 1 was noted in the presence of indigenous microbiota (compared to autoclaved controls) [91], suggesting that indigenous microbiota play an important role in the decay of infectious viruses.

Like adenovirus laboratory feeding experiments, a recent report demonstrated macropinocytosis and digestion of T4 coliphage in food vacuoles of ciliate *Tetrahymena thermophila* [92], suggesting that active virophagy by protozoans in environmental waters may be an important mechanism for viral attenuation. Similarly, ingestion by suspension feeding heterotrophic flagellates *Thaumatomonas coloniensis* and *Salpingoeca* spp. (rather than adsorption) was demonstrated for MS2 coliphage in groundwater [93]. Furthermore, some studies suggest that MS2 coliphage may be a source of nutrients for predatory protozoa [93, 94], further supporting the notion that predation may be an important biotic factor influencing viral decay. The limited number of field studies suggest that the removal of enterophages (bacteriophages infecting *Enterococcus faecalis*) [95] and F+ coliphages [96] is greater in unamended lake and river waters compared to the filtered and/or autoclaved controls, but decay of latter group appears to be subgroup specific [96]. However, a marine water in situ study showed a minimal effect of indigenous microbiota on decay of somatic and F+ coliphages, as well as GB-124 bacteriophage infecting *Bacteroides fragilis* [66], suggesting that the effect of protozoan communities on viral decay may be influenced by water type (fresh versus marine).

We observed a significant reduction of infectious adenovirus 2 and both coliphage groups (although it was more pronounced for the F+ compared to somatic coliphage) in the presence of indigenous protozoa and under the influence of ambient sunlight. This was especially pronounced after 3 to 5 days of exposure to indigenous protozoan communities, a trend that is consistent with the time required for freshwater protozoan communities to adjust to the influx of prey organisms [97–99]. This finding suggests that indigenous protozoa likely plays an important role in the decay of infectious viruses (indicators and pathogens alike), especially in freshwater habitats and in conjunction with ambient sunlight, although the magnitude of that effect is influenced by the time point and the viral target. Future studies are needed to clarify the nature of ecological interactions between protozoans and viruses and to better characterize the interplay between sunlight irradiation and impact of indigenous protozoa on viral decay.

## Conclusions

In summary, our results indicate that both somatic and F+ coliphages decay at similar rates to infectious adenoviruses in a freshwater aquatic habitat. This finding implies that their persistence in environmental waters could be similar and that coliphages may be suitable surrogates for adenovirus decay in these systems. Furthermore, while we show that the exposure to ambient sunlight plays an important role in viral decay, its effect was especially pronounced with somatic coliphages, suggesting that the mechanism of action may differ among the viruses studied. Lastly, our data suggests that protozoans play an important role in the decay of somatic and F+ coliphages and infectious adenoviruses in aquatic environments. While controlled laboratory-based studies can provide important insights into the effect of environmental factors on decay, additional field studies closely mimicking natural conditions are warranted to better characterize the interactions between indigenous protozoan communities and infectious viral pathogens and indicators.

## Data Availability

Data can be found on EPA ScienceHub website (https://catalog.data.gov/harvest/epa-sciencehub).

## Abbreviations

ANOVA: Analysis of variance
ATCC: American type culture collection
CPE: Cytopathic effects
DAL: Double agar layer
DMEM: Dulbecco’s minimum essential medium
EBSS: Earle’s balanced salt solution
FIB: Fecal indicator bacteria
MPN: Most probable number
PBS: Phosphate buffered saline
SAM: Submersible aquatic mesocosm
TSA: Tryptic soy agar
UV: Ultraviolet
